# Isolated Cembranoid Diterpenes From *Sarcophyton tenuispiculatum* and Semi‑Synthetic Derivatives with Pro‑Apoptotic Activity in Breast Cancer Cells

**DOI:** 10.1002/cbdv.71842

**Published:** 2026-10-15

**Authors:** Samin Mousavi, Afsaneh Yegdaneh, Mahmoud Aghaei, Mustafa Ghanadian

**Affiliations:** ^1^ Department of Pharmacognosy School of Pharmacy and Pharmaceutical Sciences Isfahan University of Medical Sciences Isfahan Iran; ^2^ Department of Clinical Biochemistry School of Pharmacy and Pharmaceutical Sciences Isfahan University of Medical Sciences Isfahan Iran; ^3^ Isfahan Pharmaceutical Sciences Research Center Isfahan University of Medical Sciences Isfahan Iran

**Keywords:** acute toxicity, apoptosis, breast cancer, Caspase 6, cembranoids, reactive oxygen species, *Sarcophyton*, semi‐synthesis

## Abstract

The soft coral *Sarcophyton tenuispiculatum* was investigated for its secondary metabolites, leading to the isolation of four natural isolates, including 7α,8α‐epoxy‐1(15),3,11‐cembratriene‐16,2β‐olide (**1**), 2β,16,7α,8α‐diEpoxy‐1(15),3,11‐cembratriene (**2**), apo‐9′‐fucoxanthinone (**3**), and (+)‐viridiflorol (**4**). Semi‐synthetic modification of compound **1** produced five new cembranoids: epi‐Trochelioid B (**5**), chlorobenzocembrin (**6**), benzocembrin (**7**), nitrobenzocembrin (**8**), and Trochelioid C (**9**). Structural elucidation was achieved using extensive spectroscopic analyses, including 1D/2D NMR and HR‐ESI‐MS. Biological evaluation demonstrated that chlorobenzocembrin (**6**) exhibited more cytotoxic activity against breast cancer cell lines MCF‐7 and MDA‐MB‐231, inducing apoptosis through ROS accumulation and caspase‐6 activation. Acute toxicity studies in mice revealed hepatotoxicity and nephrotoxicity in doses > 250 mg/kg. Preliminary antiproliferative SAR highlights that epoxide ring opening followed by aryl substitution at C‑7 on 7α,8α‐epoxy‐1(15),3,11‐cembratriene‐16,2β‐olide enhanced cytotoxic activity and suggests that incorporating substituents associated with enhanced cytotoxic activity at the same position may represent a promising strategy for semi‑synthetic optimization of cembranoid scaffolds toward more potent and selective anticancer agents.

## Graphical abstract

New arylated cembranoid derivatives from *Sarcophyton tenuispiculatum* show enhanced antiproliferative activity against breast cancer cells. Chlorobenzocembrin induces apoptosis through ROS accumulation and caspase‐6 activation, suggesting that epoxide ring opening and C7 aryl substitution are key structural features for strengthening anticancer potential in cembrane lactones.

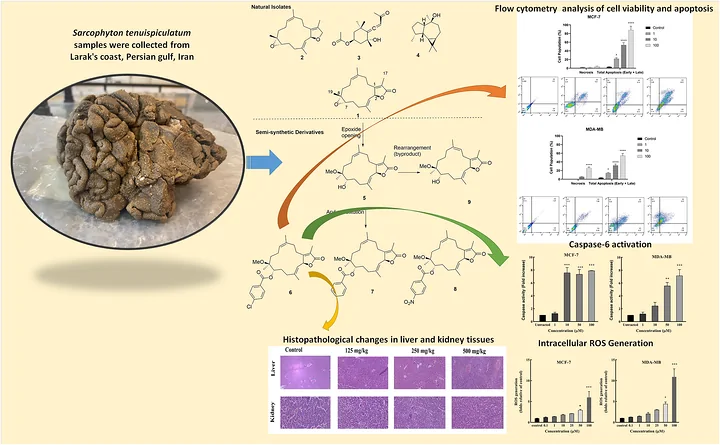

## 1 Introduction

Natural products have long been recognized as an important source of pharmaceuticals. They offer remarkable chemical diversity and have evolved over millennia. This diversity is associated with a wide range of pharmacological activities and drug‐like properties. As a result, natural products continue to serve as valuable leads in drug discovery [1]. Among these compounds, diterpenes are widely distributed in marine organisms, including marine fungi, animals, and plants. Marine‐derived diterpenes are often found in monocyclic forms [2]. Marine organisms, particularly soft corals, produce specialized secondary metabolites that help them survive in harsh environmental conditions [3]. Within this group, octocorals of the genus *Sarcophyton* (phylum Cnidaria, subphylum Anthozoa, class Octocorallia, order Malacalcyonacea, family Sarcophytidae) are broadly distributed across shallow coral reef ecosystems in the Asia‐Pacific region and are considered a rich source of bioactive natural products [4]. These metabolites include sesquiterpenes, diterpenes, steroids, and ceramides, many of which have shown anticancer, antimicrobial, anti‐inflammatory, anti‐osteoporotic, antimetastatic, antiangiogenic, and neuroprotective activities [2, 5].

In our ongoing research on chemically interesting and biologically active secondary metabolites from marine invertebrates, we examined the chemical composition of *Sarcophyton tenuispiculatum* collected from the coast of the Persian Gulf. Since cembrane compounds had been identified previously, we hypothesized that semisynthetic modification of this scaffold could alter its cytotoxic activity. Accordingly, semisynthetic modification was pursued to improve the biological profile of a naturally occurring scaffold by exploring structure‐activity relationships and introducing new substitution patterns not readily available through isolation alone (Figure 1).

**FIGURE 1:**
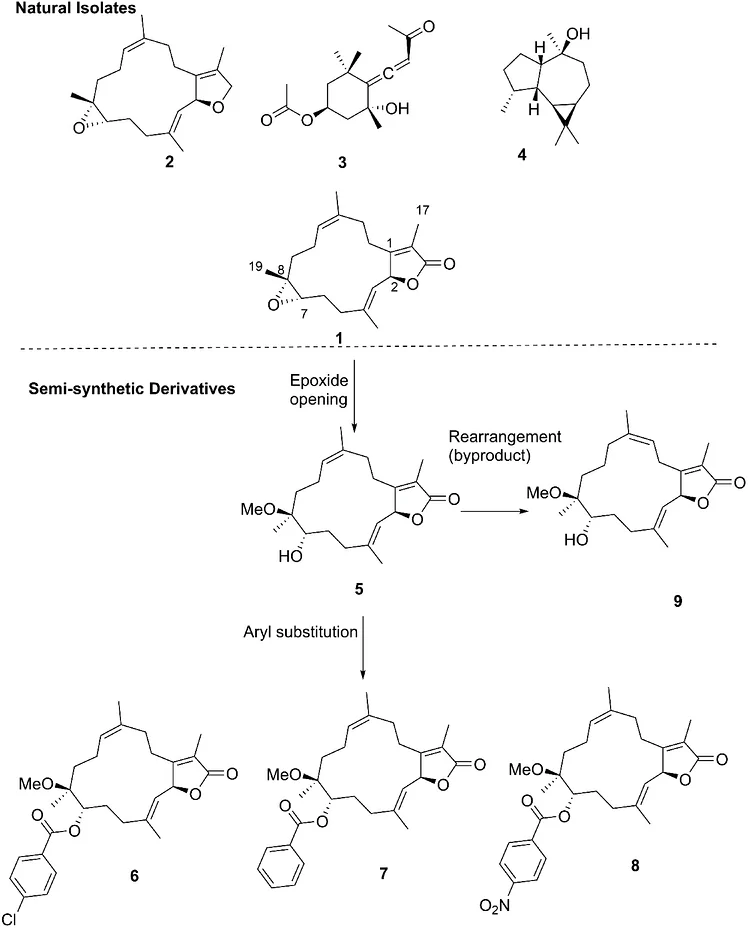
Natural isolated compounds (**1–4**) from *Sarcophyton tenuispiculatum* and semi‐synthetic derivatives of compound **1** scaffold, by epoxide ring opening (**5**), C‐7 esterification (**6–8**), and allylic double‐bond rearrangement (**9**).

The design of our semi‐synthetic compounds using sarcophine (compound **1**, 7α,8α‐epoxy‐1(15),3,11‐cembratriene‐16,2β‐olide) isolated from *S. tenuispiculatum* was based on the objective of exploring whether increased functionalization at C‐7 could improve the cytotoxic profile. This rationale was inspired by a previous chemical modification of sarcophine isolated from *Sarcophyton glaucum* [6]. In that study, Hassan and coworkers opened the epoxide ring and subsequently esterified it with a series of alkyl or aryl esters. These esterified cembranoids showed enhanced biological activity against MDA‐MB‐231 breast cancer cells compared with sarcophine. In their biological tests, compound **15**, with a benzyl carbamate/amide‐ester group at the C‐7 position, exhibited greater activity than the simple allyl esters.

## 2 Results and Discussion

### 2.1 Phytochemical Study

The coral material of *S. tenuispiculatum*, weighing a dry mass of 2.34 kg were extracted with acetone over a period of ten days at room temperature using a percolator. The resultant concentrated acetone extract was then processed through liquid‐liquid extraction using ethyl acetate and water to remove water‐soluble impurities. Subsequently, the dried ethyl acetate phase was further isolated using a separatory funnel in conjunction with methanol and hexane. The hexane fraction was rich in steroids and was put aside. In parallel, the methanol fraction underwent a series of chromatography techniques, culminating in the identification of compounds **1**‐**4**, based on ^1^H‐, ^13^C NMR and DEPT analysis, and correlations observed in HMBC and ROESY spectra and comparison with those reported in the literature.

Compounds **1** and **2** were obtained as colorless oils and identified as 7α,8α‐Epoxy‐1(15),3,11‐cembratriene‐16,2β‐olide (1) (Figures S1–S7) and 2β,16, 7α,8α‐diEpoxy‐1(15),3,11‐cembratriene, respectively (**2**) (Figures S8–S14) [4]. Compound 1 had previously been reported from *Sarcophyton glaucum* as sarcophine, a compound known for its fish‐toxic properties and potent anti‐acetylcholine activity [7, 8]. However, this is the first report of compounds **1** and **2** from *Sarcophyton tenuispiculatum*. Compound **3** was identified as apo‐9'‐fucoxanthinone (Figures S15–S20), which was first reported from the cultured marine dinoflagellate *Amphidinium* sp [9]. It has recently been demonstrated to have anti‐inflammatory activity by suppressing NO production via NF‐κB and MAPK pathways in RAW 264.7 cells, and significantly reducing ROS, NO, cell death, and pro‐inflammatory cytokines in LPS‐stimulated zebrafish, suggesting its potential as an effective anti‐inflammatory agent [10]. Compound **4** was identified as (+)‐viridiflorol (Figures S21–S24), previously reported from *Melaleuca quinquenervia* essential oil [11].

### 2.2 Epoxide Ring Opening and C‐8 Methoxylation of Compound **1**

Compound **5** was synthesized by opening the epoxide ring in compound **1** through methanolysis under acidic conditions. It was obtained as a colorless oil with the molecular formula C_21_H_32_O_4_, confirmed by (+)‐HR‐ESI‐MS with an ion peak at m/z 349.235 [𝑀+𝐻]^+^, matching the calculated value of 349.2373 (C_21_H_33_O_4_, Δ = −6.59). Carbon NMR and DEPT analysis showed 21 signals including four methyl groups: δ_C_ 9.32(C‐17), 16.68(C‐18), 18.04(C‐19), 16.06(C‐20), and one metoxy at δ_C_48.73, six methylenes: δ_C_ 36.71(C‐5), δ_C_ 28.19(C‐6), δ_C_ 31.39(C‐9), δ_C_ 21.24(C‐10), δ_C_ 38.33(C‐13), δ_C_ 26.65(C‐14), four methines including two oxygenated at δ_C_ 80.45(C‐2), and 73.59(C‐7), and two olefins at δ_C_ 117.91(C‐3), δ_C_ 127.59(C‐11), six quaternary carbons, including one oxygenated at δ_C_ 79.31(C‐8), four olefin at δ_C_163.35(C‐1), 146.62(C‐4), 135.49(C‐12), 122.86(C‐15), and one carbonyl at δ_C_ 175.15(C‐16). ^1^H NMR analysis further supported the proposed structure, showing four methyl singlets at δ_H_ 1.84 (H‐17), 1.89 (H‐18), 1.25 (H‐19), and 1.62 (H‐20), two olefinic methines at δ_H_ 4.99 (H‐3) and 5.31 (H‐11), and two oxymethines at δ_H_ 5.41 (H‐2) and 3.26 (H‐7). Compared with **1**, compound **5** showed a downfield shift of C‐7 from δ_C_ 61.84 to δ_C_ 73.59 and of C‐8 from δ_C_ 60.25 to δ_C_ 79.31, together with the appearance of a methoxy signal at δ_C_ 49.04 (δ_H_ 3.15, s, 3H), indicating epoxide ring opening between C‐7 and C‐8 and subsequent methoxylation at C‐8. This was supported by key HMBC correlations: 8‐OMe (δ_H_ 3.15) showed connectivity to C‐8(δ_C_ 79.31), and H‐7(δ_H_ 3.26) exhibited cross‐peaks with C‐6(δ_C_ 28.19) and C‐5(δ_C_ 36.71). Based on these findings, compound **5** showed similarity with 7β‐hydroxy‐8β‐methoxy‐1(15), 3,11‐cembratriene‐16,2β‐olide (Trochelioid B) isolated naturally from the Red Sea soft coral Sarcophyton trocheliophorum [12]. However, a distinct difference was observed in the orientation of H‐7, as revealed by key NOESY correlations. Based on the x‐ray crystallography of sarcophines performed by Bernstein et al., H‐2 has an α‐position. NOESY cross‐peaks of H‐2α/H‐6β, H‐6β/Me‐19, and 8‐MeO/H‐7 established the α‐orientation of H‐2 and Me‐19, together with the β‐orientation of H‐7 (Figure 2). This difference was also reflected in the ^1^H NMR data: H‐7 of compound **5** resonated at δ_H_ 3.25 (1H, dd, *J* = 1.56, 9.19 Hz), whereas H‐7 of Trochelioid B was reported at δ_H_ 3.44 (1H, d, *J* = 11 Hz) [12], indicating a different stereochemical environment at this position. Further support was obtained from the optical rotation data. Compound **5** showed [α] +14.093, whereas Trochelioid B was reported to have [α] +86.7. The difference in magnitude, together with the NOESY and coupling constant data, supports an epimerization at C‐7. On this basis, the structure was elucidated as 7α‐hydroxy‐8β‐methoxy‐1(15),3,11‐cembratriene‐16,2β‐olide as a new compound named epi‐Trochelioid B. In acidic conditions like TFA in methanol, the epoxide ring gets protonated and becomes more active. Then methanol attacks the more substituted carbon, and this leads to anti‐opening, giving trans‐oriented methoxy and hydroxy substituents at C‐8 and C‐7, respectively [13].

**FIGURE 2:**
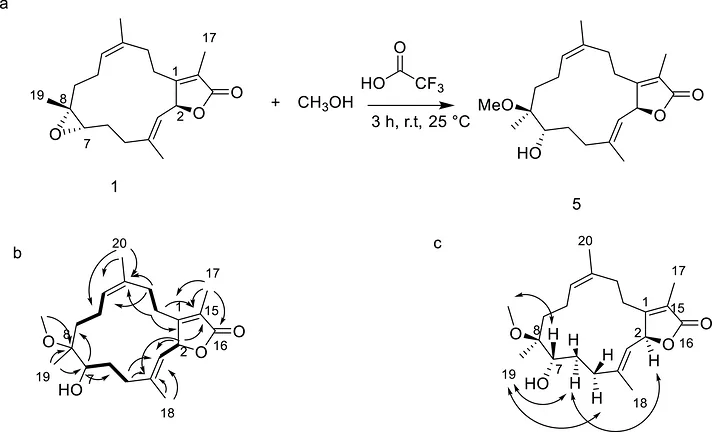
(a) Epoxide opening of 1 via methanolysis under acidic conditions. (b) Key HMBC correlations of 5, and (c) representation of key NOESY correlations of 5.

### 2.3 General Procedure for the Synthesis of Compounds **6–9**

Compounds **6**, **7**, and **8** were synthesized as arylated derivatives of compound **5**, via nucleophilic aryl substitution depending on the nature of the aryl halide. The hydroxyl group on compound **5** acts as the nucleophile, while the aryl halide serves as the electrophile. Compound **6** was obtained as a colorless oil with the molecular formula C_28_H_36_ClO_5_, as confirmed by high‐resolution electrospray ionization mass spectrometry [(+)‐HR‐ESI‐MS], which showed a molecular ion peak at m/z 487.226 ([M + H]^+^), with a mass deviation of Δ = +3.07 from the calculated value of 489.2245 for C_28_H_36_ClO_5_. The degree of unsaturation was determined to be **8**, consistent with the structural features inferred from the ^1^ ^3^C NMR data. Carbon NMR and DEPT analysis revealed 28 distinct signals, including five methyl carbons at δ_C_ 9.83(C‐17), 18.85(C‐18), 18.77(C‐19), 17.45(C‐20), and 49.27 (methoxy, C‐OMe); six methylene carbons at δ_C_ 35.58(C‐5), 25.98(C‐6), 23.44(C‐9), 33.67(C‐10), 34.99(C‐13), and 26.98(C‐14); and six methine carbons, among which two were oxygenated at δ_C_ 80.78 (C‐2) and 76.08(C‐7), and two were olefinic at δ_C_ 119.33(C‐3) and 125.61(C‐11), along with δ_C_ 131.36(C‐2′) and 129.30(C‐3′). Nine quaternary carbons were observed, including one oxygenated at δ_C_ 79.45(C‐8), four olefinic at δ_C_ 164.17(C‐1), 144.81(C‐4), 134.41(C‐12), and 123.18(C‐15), two aromatic quaternaries at δ_C_ 128.30(C‐1′) and 139.92 (C‐4′), and two carbonyl carbons at δ_C_ 175.88(C‐16) and 165.39(Ar–COO). ^1^H NMR analysis supported these assignments, showing five singlet methyl proton signals at δ_H_ 1.89(H‐17), 1.79(H‐18), 1.20(H‐19), 1.63(H‐20), and 3.25(H‐1′, methoxy). Four olefinic methine protons were detected at δ_H_ 5.02(H‐3), 5.11(H‐11), 7.95(H‐2′), and 7.42(H‐3′), while two oxymethine protons appeared at δ_H_ 5.44(H‐2) and 5.03(H‐7). Compared to the parent compound **5**, compound **6** features an additional para‐chlorobenzoyl moiety, as evidenced by the aromatic carbon signals at δ_C_ 128.30(C‐1′), 131.36(C‐2′), 129.30(C‐3′), and 139.92(C‐4′), along with the ester carbonyl at δ_C_ 165.39(Ar–COO). This substitution is located at C‐7, as indicated by the downfield shift of H‐7(δ_H_ 5.03), consistent with esterification. HMBC correlations between H‐7 and δ_C_ 165.39 further confirmed the connectivity. Based on these spectroscopic data and the reaction conditions, the structure of compound 6 was elucidated as 7α‐O‐para‐chlorobenzoyl‐8β‐methoxy‐1(15),3,11‐cembratriene‐16,2β‐olide as a new compound named chlorobenzocembrin. Similarly, compound **7** exhibited a comparable spectral profile, with minor differences attributed to the substitution of a benzoate group in place of the para‐chlorobenzoyl moiety. It was obtained with the molecular formula C_28_H_36_O_5_, confirmed by (+)‐HR‐ESI‐MS with an ion peak at m/z 453.263 (calcd 453.2635 for C_28_H_37_O_5_ [M + H]^+^), with a minor deviation of Δ = −1.1 ppm. Carbon NMR and DEPT analysis revealed 25 signals, including five methyl carbons at δ_C_ 9.76(C‐17), 19.16(C‐18), 18.88(C‐19), 17.40(C‐20), and 14.25(C‐1′), along with a methoxy carbon at δ_C_ 49.71. Eight methylene carbons were observed at δ_C_ 36.57(C‐5), 27.35(C‐6), 31.38(C‐9), 20.36(C‐10), 38.47(C‐13), 25.86 (C‐14), 18.67(C‐2′), and 37.04(C‐3′). Four methine carbons were assigned, including two oxygenated at δ_C_ 80.75(C‐2) and 74.95(C‐7), and two olefinic at δ_C_ 119.21(C‐3) and 125.46(C‐11). Seven quaternary carbons were identified, including one oxygenated at δ_C_ 79.34(C‐8), four olefinic at δ_C_ 164.12(C‐1), 144.81(C‐4), 134.20(C‐12), and 123.01(C‐15), and two carbonyl carbons at δ_C_ 175.92(C‐16) and 173.39(C‐4′). These data confirmed the structure as 7α‐O‐benzoyl‐8β‐methoxy‐1(15),3,11‐cembratriene‐16,2β‐olide, as a new compound named benzocembrin. Compound **8** was prepared in the same way. It was obtained with molecular formula: C_28_H_35_NO_7_, confirmed by (+)‐HR‐ESI‐MS with an ion peak at m/z 498. 453.263 (calcd 498.2486 for C_28_H_36_NO_7_ [M + H]^+^, with a minor deviation of Δ = −3.41 ppm). Carbon NMR and DEPT analysis revealed 28 distinct signals. These included five methyl carbons at δ_C_ 9.76(C‐17), 19.16(C‐18), 18.88(C‐19), 17.40(C‐20), and 14.25(C‐1′), along with a methoxy carbon at δ_C_ 49.71. Eight methylene carbons were observed at δ_C_ 36.57(C‐5), 27.35(C‐6), 31.38(C‐9), 20.36(C‐10), 38.47(C‐13), 25.86(C‐14), 18.67(C‐2′), and 37.04(C‐3′). Four methine carbons were assigned, including two oxygenated at δ_C_ 80.75(C‐2) and 74.95(C‐7), and two olefinic at δ_C_ 119.21(C‐3) and 125.46(C‐11). Seven quaternary carbons were identified, comprising one oxygenated at δ_C_ 79.34(C‐8), four olefinic at δ_C_ 164.12(C‐1), 144.81(C‐4), 134.20(C‐12), and 123.01(C‐15), and two carbonyl carbons at δ_C_ 175.92(C‐16) and 173.39 (C‐4′). Compared to compound **6**, compound **8** differs by the presence of a para‐nitrobenzoyl moiety at the C‐7 position, replacing the para‐chlorobenzoyl group. It was elucidated as 7α‐O‐paranitrobenzoyl‐8β‐methoxy‐1(15),3,11‐cembratriene‐16,2β‐olide as a new compound named nitrobenzocembrin. Compound **9** was isolated as an intermediate or byproduct during the synthesis of compound **8**. It was characterized with the molecular formula C_21_H_32_O_4_, as confirmed by (+)‐HR‐ESI‐MS, which exhibited an ion peak at m/z 349.237 [M + H]^+^ (calculated for C_21_H_28_O_4_+H^+^: 349.2373) with 6° of unsaturation. ^1^ ^3^C NMR and DEPT analysis revealed 21 carbon signals, including four methyl groups at δ_C_ 8.74(C‐17), δ_C_ 16.23(C‐18), δ_C_ 18.45(C‐19), and δ_C_ 15.65(C‐20), along with one methoxy group at δ_C_ 49.20. Six methylene carbons were observed at δ_C_ 35.50(C‐5), δ_C_ 26.73(C‐6), δ_C_ 30.96(C‐9), δ_C_ 23.29(C‐10), δ_C_ 36.04(C‐11), and δ_C_ 26.52(C‐14). Four methine carbons were identified, including two oxygenated centers at δ_C_ 79.49(C‐2) and δ_C_ 72.80(C‐7), and two olefinic carbons at δ_C_ 120.88(C‐3) and δ_C_ 125.38(C‐13). Additionally, six quaternary carbons were assigned, comprising one oxygenated carbon at δ_C_ 79.08(C‐8), four olefinic carbons at δ_C_ 162.92(C‐1), δ_C_ 144.14(C‐4), δ_C_ 134.35(C‐12), and δ_C_ 122.74(C‐15), as well as two carbonyl carbons at δ_C_ 174.95(C‐16). The ^1^H NMR spectrum displayed five singlet methyl proton signals at δ_H_ 1.81 (H‐17), δ_H_ 1.84(H‐18), δ_H_ 1.09(H‐19), and δ_H_ 1.63(H‐20), along with a methoxy proton at δ_H_ 3.17. Two olefinic methine protons were observed at δ_H_ 4.93(H‐3) and δ_H_ 5.07(H‐13), while two oxymethine protons appeared at δ_H_ 5.56(H‐2) and δ_H_ 3.43(H‐7). Comparison with compound **5** revealed a key structural difference in the position of the olefinic bond between C‐12 and C‐13. HMBC correlations substantiated this revised connectivity: Me‐20 showed cross‐peaks to δ_C_ 134.35(C‐12) and δ_C_ 125.38(C‐13), consistent with the repositioned double bond. Proton H‐11 exhibited long‐range correlations to δ_C_ 134.35(C‐12) and δ_C_ 36.90(C‐13), while H‐13 coupled to δ_C_ 36.04(C‐11), further supporting the shift in unsaturation. Additionally, H‐2 correlated with δ_C_ 26.52(C‐13), reinforcing the new olefinic arrangement. These spectral features and key HMBC interactions collectively confirmed the relocation of the double bond from the C‐11/C‐12 position to between C‐12 and C‐13. Accordingly, compound **9** was elucidated as 7α‐hydroxy‐8β‐methoxy‐1(15),3,12‐cembratriene‐16,2β‐olide, a novel cembranoid diterpene, and was designated Trochelioid C (Figure 3).

**FIGURE 3:**
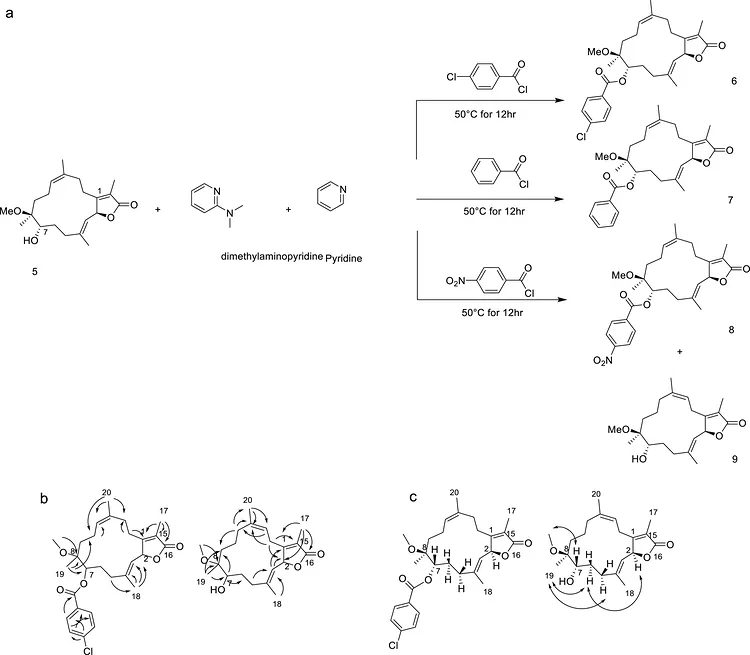
(a) Semi‑synthetic arylated derivatives (**6–9**) prepared from compound **5**. (b) Key HMBC correlations of **6** and **9**, and (c) representation of key NOESY correlations of **6** and **9**.

### 2.4 Cell Proliferation Assay

The cytotoxic activities were compared across two breast cancer cell lines: MCF‐7 and MDA‐MB. In comparison between cembranoid derivatives, Compound **1** exhibited no cytotoxicity in the MCF‐7 cell line (IC_50_> 200 µM), but minimal cytotoxicity in MDA‐MB (IC_50_ = 198 µM). Compounds **5** and **9** similarly showed negligible activity in both lines, with IC_50_ values near 200 µM. Compound **2** demonstrated selective cytotoxicity toward MDA‐MB cells (IC_50_ = 147.9 µM), while remaining less active in MCF‐7. Compound **6** displayed moderate potency in MDA‐MB cells (IC_50_ = 38.10 µM) and MCF‐7 (IC_50_ = 77.44 µM). Compound **7** showed less cytotoxic activity in MCF‐7 cells (IC_50_ = 152.5 µM) compared to MDA‐MB (IC_50_ = 138.7 µM). Compound **8** was less active than **7** in both cells. Overall, MDA‐MB cells appeared more susceptible to the tested compounds, with lower IC_50_ values observed for compounds **2**, **6**, **7**, **8**, and **9**. In none cembraboid compounds, compound **3** showed negligible activity in both cell lines (IC_50_>200 µM). Compound **4** showed more activity in MDA‐MB (IC_50_ = 144.03 µM) than MCF‐7 (IC_50_ = 198 µM). Taxol was used as a positive control. It showed cytotoxicity in both lines, with IC_50_ = 1.57 ± 0.23 µM in MCF‐7 and 0.62 ± 0.07 µM in MDA‐MB (Table 1, Figure 4).

**TABLE 1:** IC_50_ values of compounds **1‐9** against MCF‐7 and MDA‐MB‐231 breast cancer cells.

Compound	MCF‐7 (µM)	MDA‐MB (µM)
**1**	>200	198.3 ± 16.8
**2**	>200	147.9 ± 8.6
**3**	>200	>200
**4**	144.03 ± 10.5	198.1 ± 13.6
**5**	>200	>200
**6**	77.44 ± 4.8	38.10 ± 2.4
**7**	152 ± 9.1	138.7 ± 8.2
**8**	>200	150.1 ± 9.4
**9**	197.2 ± 13.5	189 ± 11
Taxol	1.57 ± 0.2	0.62 ± 0.1

*Note*: Data are expressed as mean of IC_50_ values ± SD (*n* = 3).

**FIGURE 4:**
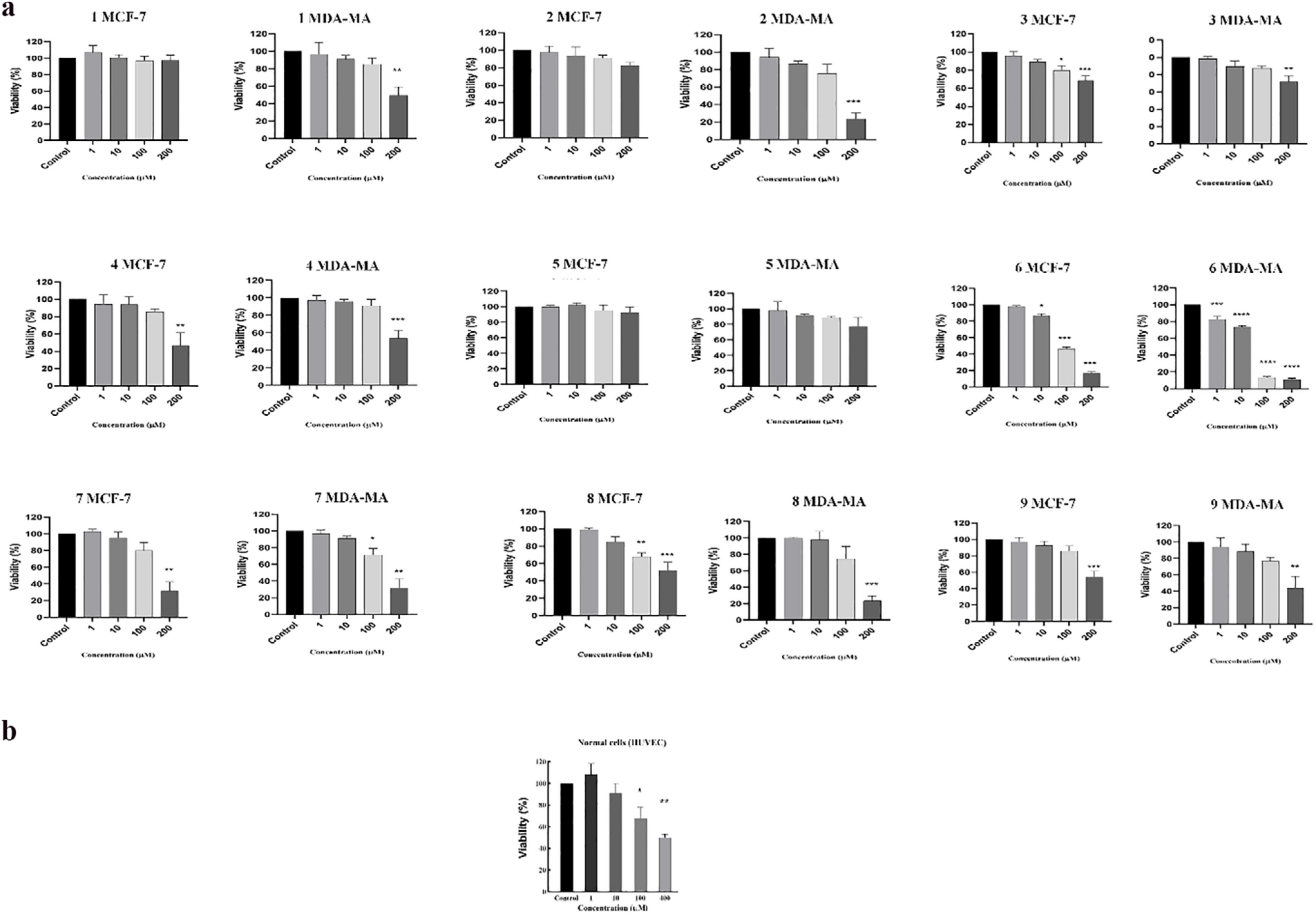
Cytotoxic effects of compounds **1**–**9**. (a) Cytotoxic effects against MCF‑7 and MDA‑MB‑231 breast cancer cells (MTT assay). (b) Cytotoxic effect of compound **6** against HUVEC normal cells. Statistical significance was determined via one‐way ANOVA, with *P* < 0.05 (*), *P* < 0.01 (**), *P* < 0.001 (***), and *P* < 0.0001 (****), indicating significant differences compared to untreated control cells (*N* = 3).

Compound **6**, as a selective compound was tested also against normal cells (HUVEC cell line) with IC_50_ values of 356.5 ± 34.6 µM. Its selectivity index, as the ratio of normal cell IC_50_ to that of cancer cells, was calculated as 3.66 for MCF‐7 and 7.44 for MDA‐MB cells, indicating greater selectivity toward MDA‐MB cells.

Preliminary SAR analysis showed that opening the epoxide ring and adding an aryl group at C‑7 increased cytotoxicity. This was seen in compounds 6 and 7 compared to the parent scaffold (1). In contrast, Ent‑Trochelioid B (5) and Trochelioid C (9), both derived from epoxide opening and C‑8 methoxylation, were essentially inactive. This suggests that epoxide opening alone, or shifting the olefin within the macrocycle, does not increase cytotoxic activity. Likewise, comparison of sarcophine (1) with its di‐epoxy analogue (2) revealed only a slight reduction in IC_50_ against MDA‐MB‐231 cells, without meaningful improvement overall. These findings highlight the importance of C‑7 esteric substitution and suggest that incorporating more cytotoxic substituents at this position may serve as a promising strategy for new cembranoid scaffolds with more potent and selective anticancer agents. Moreover, a comparison of the electronic effects of the para‐substituents revealed that the functionalization of the C‐7 benzoyl group significantly influenced cytotoxic activity. Compound 6 with para‐chloro showed the highest activity, suggesting that this group is favorable for activity. In contrast, compound 8 with para‐nitro showed much lower activity, indicating that a strong electron‐withdrawing group is not favorable at this position. These results suggest that the electronic nature of the para‐substituent is important for activity.

### 2.5 Flow Cytometric Analysis of Apoptosis

Compound **6** (chlorobenzocembrin) showed more cytotoxic activity than other cembranoid derivatives, so it was chosen for further mechanistic study. Flow cytometry was used to analyze apoptosis and necrosis caused by chlorobenzocembrin in MCF‐7 and MDA‐MB cell lines. Annexin V‐FITC and propidium iodide (PI) staining were applied. Dot plots showed cell distribution across four quadrants, with Q4 containing viable cells (Annexin^−^/PI^−^); Q3, early apoptotic cells (Annexin^+^/PI^−^); Q2, late apoptotic cells (Annexin^+^/PI^+^); and Q1, necrotic cells (Annexin^−^/PI^+^). In both cell lines, chlorobenzocembrin increased apoptosis in a dose‐dependent way. In MCF‐7 cells, low doses (1 and 10 µM) mainly caused early apoptosis, with minimal necrosis. At the highest concentration (100 µM), a shift toward late apoptosis was seen, along with a significant decline in viable cell populations. MDA‐MB cells showed a similar pattern. But at 100 µM, they had more necrosis than MCF‐7. Overall, chlorobenzocembrin triggers dose‐dependent apoptosis in both cell lines. At higher concentrations, MCF‐7 cells favored apoptosis, while MDA‐MB cells showed a mixed apoptosis–necrosis profile (Figure 5).

**FIGURE 5:**
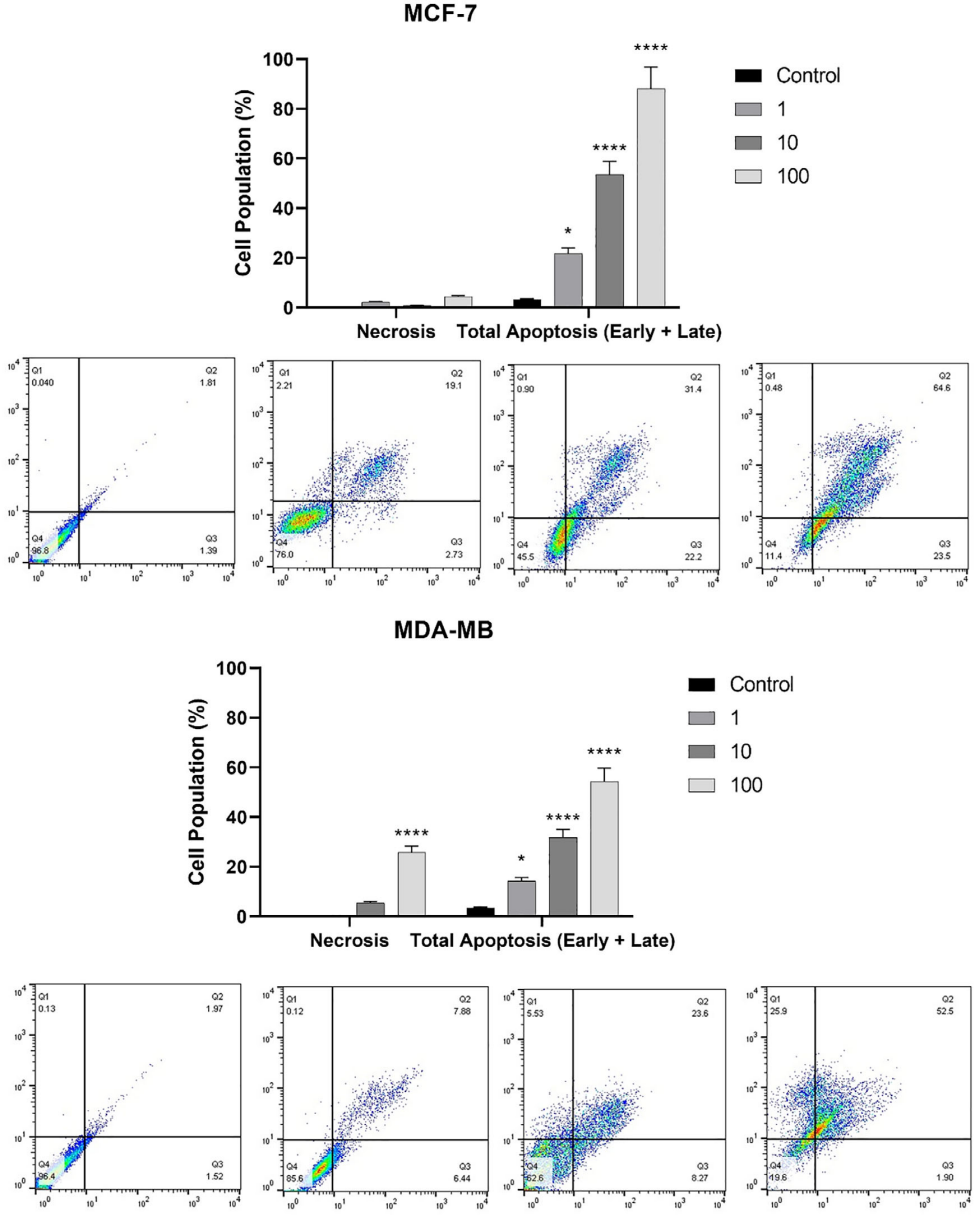
Flow cytometric analysis of apoptosis in MCF‐7 and MDA‐MB‐231 cells treated with chlorobenzocembrin. Quadrant analysis of dot plots identified viable cells in Q4, early apoptotic cells in Q3, necrotic cells in Q1, and late apoptotic cells in Q2. Statistical significance compared to untreated controls was denoted as follows: *P < 0.05, **P < 0.01, ***P < 0.001, ****P < 0.0001. Data were analyzed using one‐way ANOVA.

### 2.6 Caspase‐6 Activation in Breast Cancer Cells Following Chlorobenzocembrin Treatment

Due to the genetic deficiency of caspase‐3 expression in the MCF‐7 cell line, downstream executioner caspase activation was evaluated using a Caspase‐6 Activity Assay Kit. Briefly, cells were exposed for 48 h to increasing concentrations (1, 10, 50, and 100 µmol/L). Both cell lines showed dose‐dependent caspase activation, but with different sensitivities. In MCF‐7 cells, caspase‐6 activity was significantly increased at a concentration of 10 µmol/L. It increased further at 50 and 100 µmol/L, indicating significant apoptotic induction. In the same way, MDA‐MB cells displayed a gradual response. They showed moderate activation at 50 µmol/L and maximum activity at 100 µmol/L. At 1 µmol/L, neither cell line showed significant changes. These results confirmed caspase‐6 activation, but MCF‐7 cells were more sensitive at lower doses than MDA‐MB cells (Figure 6).

**FIGURE 6:**
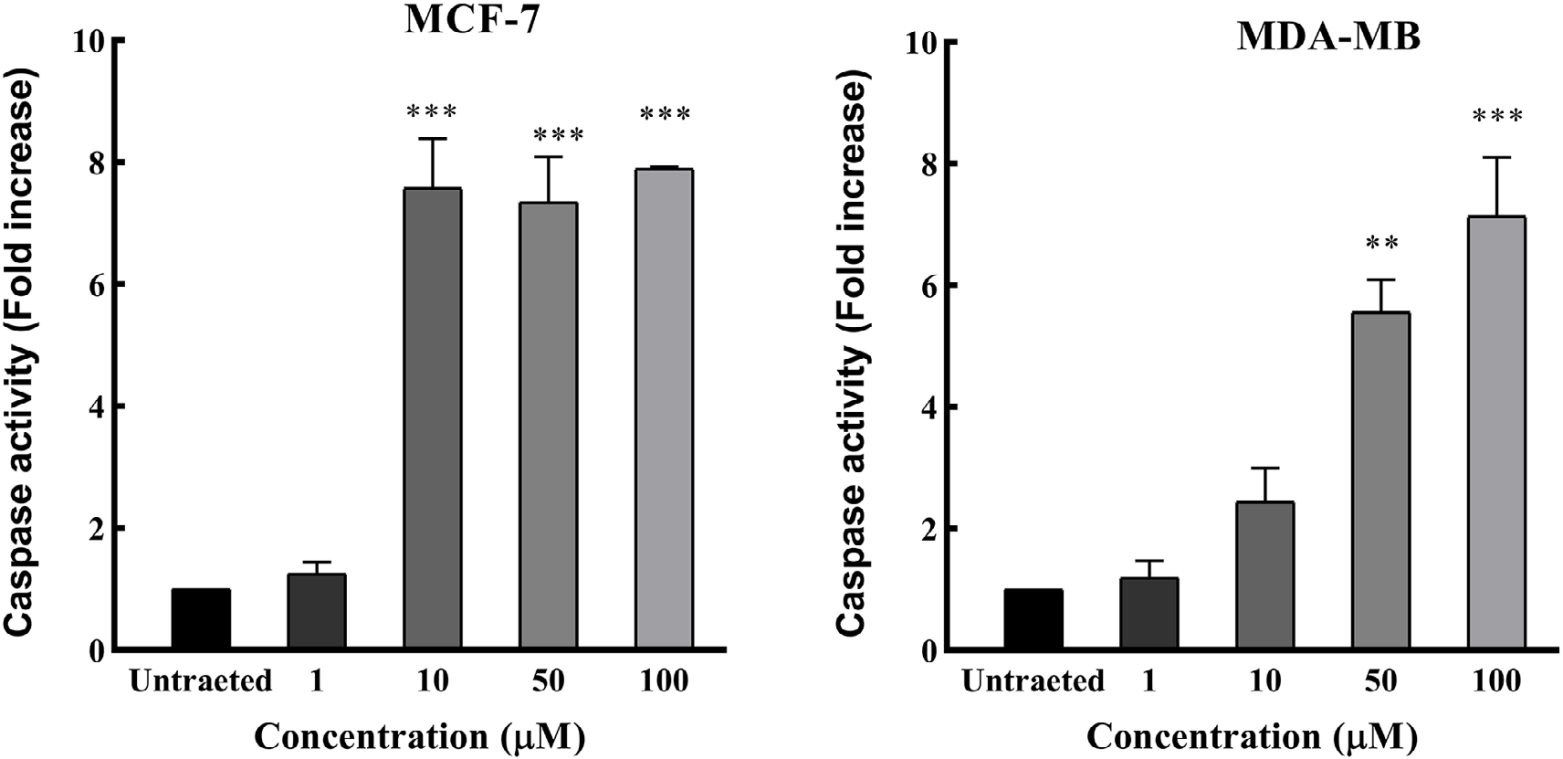
Dose‑dependent caspase‑6 activation in MCF‑7 and MDA‑MB‑231 cells following chlorobenzocembrin exposure. *P < 0.05, **P < 0.01, and ***P < 0.001 indicate statistically significant differences compared to control cells. Statistical analysis was performed using one‐way ANOVA. All experiments were conducted in triplicate.

### 2.7 Intracellular ROS Generation Assay

The effect of chlorobenzocembrin on intracellular reactive oxygen species (ROS) generation was measured against MCF‐7 and MDA‐MB breast cancer cells using the DCFH‐DA fluorescence assay. Cells were treated with increasing concentrations of chlorobenzocembrin (1, 10, 50, and 100 µmol/L) for 48 h, and ROS levels were quantified relative to untreated controls. Both cell lines showed a dose‐dependent rise in ROS. In MCF‐7 cells, ROS levels remained non‐significant up to 25 µmol/L. At 50 µmol/L, activation was significant with a p‐value < 0.05. At 100 µmol/L, the increase was more significant with a *p*‐value < 0.001. MDA‐MB cells showed a similar pattern. ROS rose significantly at 50 µmol/L, and maximum induction occurred at 100 µmol/L with a *p*‐value < 0.01. These findings show that chlorobenzocembrin triggers oxidative stress in a concentration‐dependent way, but MCF‐7 cells are more sensitive to this drug (Figure 7).

**FIGURE 7:**
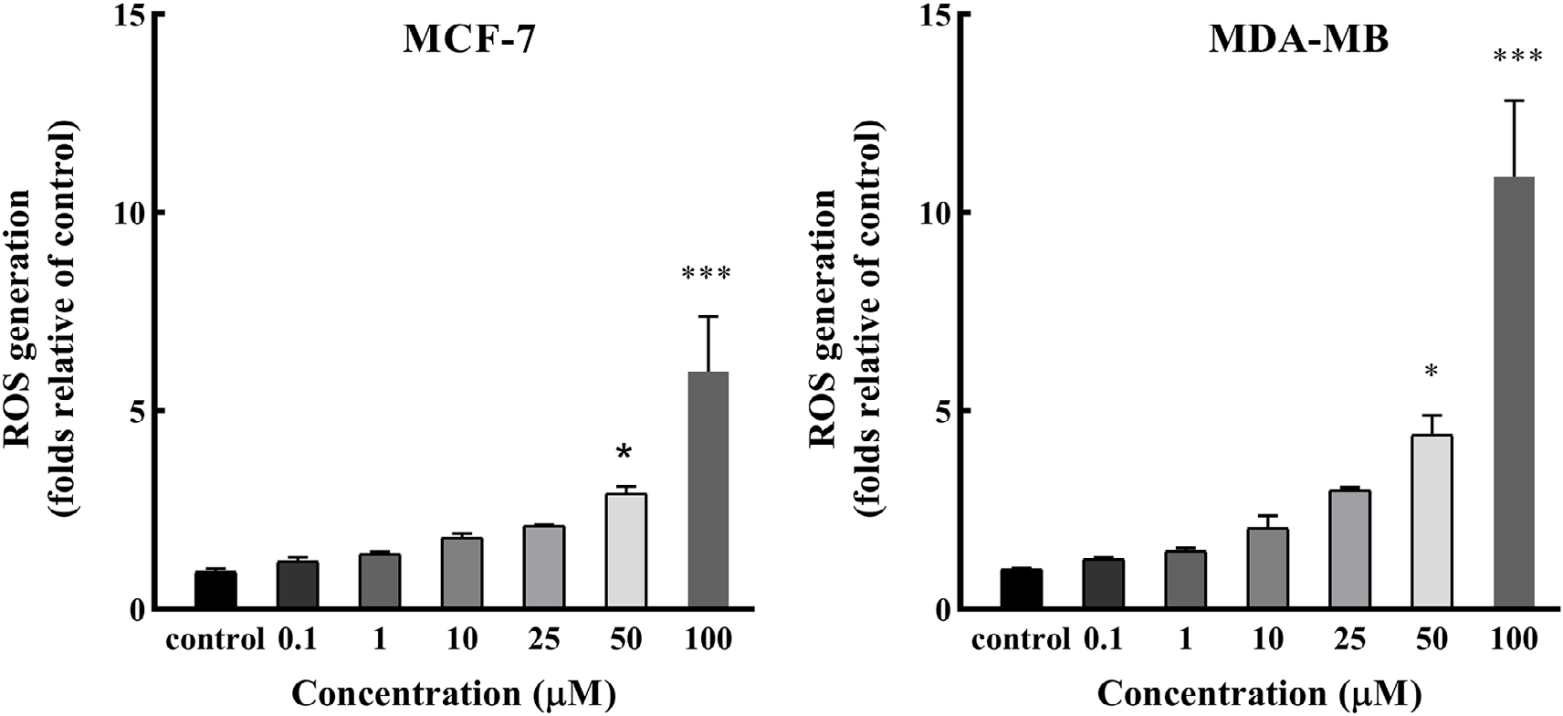
Intracellular ROS generation in MCF‑7 and MDA‑MB‑231 cells induced by chlorobenzocembrin. Cells were treated with increasing concentrations of chlorobenzocembrin (1–100 µmol/L) for 48 h, followed by incubation with 2’,7’‐dichlorofluorescein diacetate. Fluorescence intensity was measured using a microplate reader, and ROS levels were expressed as fold change relative to untreated controls (mean ± SD). *P < 0.05, **P < 0.01, and *P* < 0.001 indicate statistically significant differences compared to control cells. Statistical analysis was performed using one‐way ANOVA. All experiments were conducted in triplicate.

### 2.8 Acute Toxicity and Liver and Kidney Following Acute Exposure to Graded Doses of Chlorobenzocembrin in Mice

#### 2.8.1 Histopathology

The 14 days acute toxicity study was conducted on chlorobenzocembrin in mice. Animals were kept under controlled laboratory conditions. They received oral doses of 125, 250, and 500 mg/kg. A vehicle control group was included. All mice were fasted before treatment. They were monitored for clinical signs, body weight, and survival over 14‐days. Within the first 24 h, surviving animals appeared hunched, with contracted bodies, closed eyes, and little movement. No convulsions were seen. After 24 h, these mice recovered, showing normal activity and mobility. Surviving mice on day 15 were euthanized and checked for possible liver and kidney toxicity. Histological examination of the liver revealed a dose‑dependent pattern of hepatotoxicity at the highest concentration. Control animals showed normal liver structure, intact hepatocytes, and clear sinusoids. At 125 mg/kg, mild vacuolation and slight sinusoidal dilation appeared, with minimal vascular congestion. At 250 mg/kg, some hepatocyte ballooning and focal sinusoidal disruption were seen. At 500 mg/kg, more balloonings, necrosis, structural disorganizations, and vascular damages were present (Figure 8). Kidney histology also showed a dose‐dependent nephrotoxicity. Vehicle‐treated control mice exhibited normal renal architecture with intact tubular epithelium and preserved glomeruli. At 125 mg/kg, mild tubular swelling and slight mesangial expansion, with minimal vascular congestion, appeared. At 250 mg/kg, the kidneys showed some tubular degeneration and glomerular shrinkage. At 500 mg/kg, more tubular necrosis, glomerular collapses, and vascular damage were seen (Figure 8).

**FIGURE 8:**
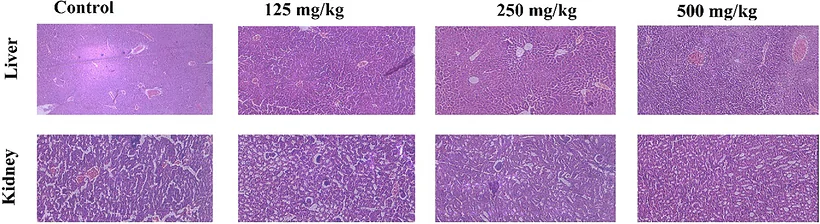
Histopathological changes in liver and kidney tissues of mice in acute toxicity test, after 14‑day single i.p. administration of chlorobenzocembrin (control, 125, 250, and 500 mg/kg). Liver sections show dose‐dependent hepatotoxic changes, ranging from mild vacuolation and sinusoidal dilation at 125 mg/kg to severe ballooning, necrosis, and inflammatory infiltration at 500 mg/kg. Kidney sections reveal progressive tubular and glomerular damage, with mild swelling at 125 mg/kg and marked necrosis and vascular injury at 500 mg/kg. All samples were technically adequate and included in the analysis.

Overall, the results reveal a clear dose‐response relationship, with both the liver and kidneys as primary targets of toxicity. At the lowest tested dose (125 mg/kg), the observed clinical signs were transient, with full behavioral recovery observed within 24 h. This, coupled with the absence of permanent structural damage in histopathological examinations (showing only mild, reversible changes like vacuolation), indicates that the systemic stress at this level was acute yet reversible during the 14‐day observation period.

#### 2.8.2 Effects on Serum Biochemical Parameters

Chlorobenzocembrin administration caused dose‐dependent changes in the kidney and liver. In the kidney, serum sodium and potassium dropped steadily dose‐dependently, from control to 500 mg/kg. This indicated increased electrolyte loss and impaired tubular reabsorption at higher doses, consistent with worsening tubular and glomerular injury at these doses.

In the liver, total protein and albumin declined gradually but without significant suppression of total protein synthesis. In contrast, alkaline phosphatase (AP) and alanine aminotransferase (AST) rose sharply at 500 mg/kg, while aspartate aminotransferase (ALT) showed a moderate increase (Table 2).

**TABLE 2:** Effects of Chlorobenzocembrin on Kidney and Liver Serum Parameters in an acute toxicity test, after 14‑day single i.p. administration of chlorobenzocembrin (control, 125, 250, and 500 mg/kg).

Groups	Total protein (g/dl)	Albumin (g/dl)	AP (IUl)	SGOT (AST) (IU/l)	SGPT (ALT) (IU/l)	Sodium (mEql/l)	Potassium (mEq/l)
Vehicle treated	6.3 ± 0.19	3.8 ± 0.14	374 ± 10.5	119 ± 7.8	62 ± 2.7	171 ± 8.7	6.5 ± 0.34
125 mg/kg	5.3 ± 0.15	3.5 ± 0.13	489 ± 13.1	109 ± 8.5	81 ± 2.8	156 ± 7.2	5.5 ± 0.25
250 mg/kg	5.2 ± 0.19	3.3 ± 0.15	578 ± 13.8	130.1 ± 12.1	82.6 ± 3.4	154 ± 6.7	5.3 ± 0.21
500 mg/kg	5.1 ± 0.18	3.4 ± 0.16	623 ± 14.6	189 ± 14.4	120.1 ± 5.2	150 ± 7.8	4.9 ± 0.23

## 3 Materials and Methods

### 3.1 Sample Material

*Sarcophyton tenuispiculatum* samples were collected from Larak's coast, Bushehr Province, Iran, in September 2022, at a depth of 12 meters, and preserved at −20°C until use. Dr. Melika Nazemi, a Marine taxonomist, identified the coral, along with a sample bearing voucher specimen No. SAM‐4139 is deposited in the Samsam‐Shariat herbarium, Department of Pharmacognosy, Isfahan University of Medical Sciences.

### 3.2 Extraction and Purification

Coral bodies of *S. tenuispiculatum* (wet weight 17 kg) were directly suspended in methanol, following manual squeezing of the coral tissue to remove water effectively. Then, the materials were chopped into small fragments using gardening scissors to facilitate drying. These fragments were left at room temperature (20°C–25°C) overnight until completely dried, yielding 2.34 kg of dry coral matter. The sample was extracted using acetone (12 L) at a flow rate of 7 mL/min for up to ten days at ambient temperature using a percolator. The *S. tenuispiculatum* extract (STE) was filtered and then evaporated under vacuum using a rotary evaporator (concentrated extract: 342 g) and finally refrigerated at −20°C for subsequent analysis. The concentrated acetone extract was subjected to liquid–liquid separation between ethyl acetate and water to remove water‐soluble impurities. Dried ethyl acetate phase (237 g) was partitioned again using a separation funnel with methanol and hexane. The hexane fraction (67 g) was subjected to vacuum column chromatography (VLC) (7 × 20 cm; 40‐60 µm; 500 g) using a gradient of hexane: ethyl acetate solvent system: 100:0 (1.5 L, ST‐1‐1), 97:3 (1.5 L, ST‐1‐2), 92:8 (2 L, ST‐1‐3), and 88:12 (2 L, ST‐1‐4). ST‐1‐1 (52.7 g), analyzed by GC‐MS after silylation, was mainly composed of long‐chain fatty esters. ST‐1‐2 (8.1 g) consisted of a pure oil identified as compound **1** with a cembrane structure. Fractions ST‐1‐3 and ST‐7‐4 (collectively 8.9 g) were rich in steroids and were submitted for further evaluation of their antimicrobial properties. Similarly, the methanol fraction (106 g) was subjected to column chromatography (7 × 20 cm; 40‐60 µm; 500 g) using a stepwise gradient of hexane: ethyl acetate: 92:8 (2.5 L, ST‐2‐1); 87:13 (2.5 L, ST‐2‐2); 82:18 (2.5 L, ST‐2‐3); 77:23 (2.5 L, ST‐2‐4). ST‐2‐2 (0.8 g) was submitted on CC (2 × 80 cm; 40‐60 µm; 150 g) employing hexane: ethyl acetate: 100:0 (200 mL, ST‐3‐1) and 95:5 (200 mL, ST‐3‐2). ST‐3‐2 was identified as compound **2** (23 mg) with a sesquiterpene structure. ST‐2‐3 (5.4 g) consisted of a semi‐pure oil with a cembrane structure. 45 mg of sample was injected into a Waters HPLC equipped with a YMC silica column (2 × 25 cm; 10 µm) using hexane: ethyl acetate (85:15) at 3 mL/min and yielded compound **3** (ST‐4‐1) at Rt = 42 min. A portion of ST‐2‐4 (2.1 g) was purified using open column chromatography (2 × 80 cm, 40–60 µm, 106 g) and a gradient of chloroform: ethyl acetate: methanol 95:5:0 (270 mL, ST‐5‐1); 90:10:0 (270 mL, ST‐5‐2); 84:15:1 (270 mL, ST‐5‐3); 77:22:1 (270 mL, ST‐5‐4). ST‐5‐4 was more purified on an HPLC system with Shimpack RP‐18 column (2.5 × 25 cm; 5 µm) using methanol: water (70:30) at 3 mL/min and yielded compound 4 (ST‐6‐1, 5.6 mg) at Rt = 51 min.

### 3.3 Epoxide Ring‐Opening and Methylation Procedure

For the epoxide ring‐opening and methylation procedure, compound **1** (100 mg) was dissolved in 4 mL of methanol. Allow the reaction to stir for 45 min at room temperature on a magnetic shaker in an ice bath. After 50 min under nitrogen, slowly add 1 mL of TFA. After 15 min of stirring, add another 1 mL of TFA. After 1 h, monitor the reaction progress by TLC (chloroform: methanol: DEA: water, 93:5:0.15: 1). After the reaction was complete, 10 mL of saturated brine was added to the ice bath to quench the reaction and partition the mixture with chloroform to obtain compound 5 in a pure state (92 mg) [14].

**7α‐hydroxy‐8β‐methoxy‐1(15),3,11‐cembratriene‐16,2β‐olide (epi‐Trochelioid B, 5)**: colorless oil; 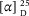
+14.093 (*c* 10.7 EtOAc); IR (KBr) ν_max_ 3465, 2933, 2858, 1732, 1674, 1446, 1373, 1304, 1217, 1173, 1095, 997 cm^−1^; for ^1^H and ^13^C NMR data, see Table 3; UV (CHCl_3_) λ_max (log ε) 246(0.67); HR‐ESI‐MS *m/z* 349.235, calcd. for C_21_H_32_O_4_ + H^+^, 349.2373(Figures S25–S31).

**TABLE 3:** ^1^H and ^13^C NMR data of compounds **5–8** (CDCl_3_, 400 MHz for δ_H_; 100 MHz for δ_C_).

	5		6		7		8	
Atom	δ_H_ (mult., *J* in Hz)	δ_C_	δ_H_ (mult., *J* in Hz)	δ_C_	δ_H_ (mult., *J* in Hz)	δ_C_	δ_H_ (mult., *J* in Hz)	δ_C_
1	—	163.35	—	164.17	—	163.98	—	164.10
2	5.4 (1H, *dd*, *J* = 10.01,1.01 Hz)	80.45	5.47 (1H, *dd,J* = 1.41, 9.60)	80.78	5.48 (1H, *d*, *J* = 9.38 Hz)	80.49	5.47(1H, *dd,J* = 1.94, 9.69)	80.49
3	4.93 (1H, *d*, *J* = 10.01 Hz)	117.91	5.02 (1H,*dd*,*J* = 1.17, 9.64)	119.33	5.05 (1H, *dd*, *J =* 1.41, 9.8 Hz)	118.90	4.93 (1H, *dd*,*J* = 1.38, 9.61)	119.22
4	—	146.35	—	144.82	—	144.67	—	144.21
5	2.41(1H, *m*),2.29(1H, *m*)	36.71	2.32(1H, *s*), 2.40 (1H, *m*)	35.58	2.29 (1H,*m*), 2.03(1H,*m*)	35.24	2.38(1H, *s*), 2.28 (1H, *m*)	35.25
6	1.80(1H, *m)*, 153(1H, *m*)	28.19	2.64 (2H,*m*)	25.98	2.63(1H,m), 2.55 (1H, *d*, *J* = 3.88)	26.69	2.63(1H,m), 2.47 (1H, *m*)	25.73
7	3.25 (1H, *dd*, *J* = 1.56, 9.19 Hz)	73.59	5.22 (1H, *dd*, *J* = 4.63, 7.82)	76.10	5.23(1H, *td*, *J* = 2.25, 4.63)	75.35	5.28 (1H,*dd*, *J* = 4.75, 7.75)	76.08
8	—	79.31	—	79.45		79.29	—	79.08
9	1.55 (1H, *dd*, *J* = 3.4, 1.3 Hz)	31.39	2.17 (2H, *m*)	23.44	1.89 (2H,*d, J = 1.8*)	25.73	2.16(1H, *d*, *J* = 3.55)	23.07
10	1.98(1H, *m*), 2.12(1H, *m*)	21.24	1.74(1H, *m*), 1.67(1H, *m*)	33.67	1.73 (1H, *m)*	33.61	1.75(*m*, 1H) 1.64(*t*, *J* = 1.25, 1H)	33.22
11	5.32 (1H, *t*, *J* = 7.5 Hz)	127.59	5.08 (1H, *t, J = 6.46, 13.08*)	125.61	5.09 (1H, *t, J =* 6.57, 13.20)	125.36	5.07 (1H, *t, J = 6.46, 13.08*)	125.23
12	—	135.49	—	134.41	—	134.00	—	134.20
13	2.17(1H, *m*), 2.34(1H, *m*)	38.33	2.40 (1H, *m*), 1.98(1H,*m*)	34.99	2.34 (1H, *m*), 2.04 (1H, *m*)	34.77	2.39 (1H, *m*), 1.95 (1H,*m*)	34.83
14	2.69 (1H, *m*), 1.96(1H, *m*)	26.65	2.17 (1H,*m*),1.82 (1H, *m*)	26.98	2.10(1H, *m*),1.82 (1H, *m*)	30.28	2.11 (1H,*m*),1.83 (1H, *m*)	26.62
15	—	122.86	—	123.18	—	122.78	—	122.98
16	—	175.15	—	175.88	—	175.81	—	175.74
17	1.89(3H,*t, J =* 1.64 Hz)	9.32	1.89 (3H, *t*, *J* = 1.69 Hz)	9.83	1.89(3H, *s*)	9.50	1.90(3H, *s*)	9.59
18	1.95 (3H, *d*, *J* = 1.63)	16.68	1.79 (3H, *s*)	18.85	1.88 (3H,*d*, *J* = 1.75)	23.13	1.21(3H, *d*, *J* = 1.38)	18.53
19	1.63 (3H, *s*)	18.04	1.2 (3H, *s*)	18.77	1.20 (3H, *s*)	18.52	1.79(3H, *s*)	18.46
20	1.06(3H, *s*)	16.04	1.63 (3H, *s*)	17.45	1.61 (3H, *s*)	17.12	1.64(3H, *s*)	17.17
8‐OMe	3.16 (3H, *s*)	48.72	3.25(3H, *s*)	49.27	3.25(3H, *s*)	49.63	3.25(3H, *s*)	49.49
7‐O‐RO	—		—	165.30	—	165.88	—	168.53
1′	—		—	128.30	—	130.61	—	135.94
2′	—		7.95(1H, *d, J =* 8.6)	131.36	8.02 (1H, *d, J =* 7.2)	129.67	8.31(2H, *d, J =* 8.8)	130.80
3′	—		7.42(1H, *d*, *J* = 8.5)	129.30	7.45 (2H, *t*, *J* = 1.56,5.50,7.38)	128.61	8.19(2H, *d*, *J* = 8.9)	122.98
4′	—		—	139.92	7.57 (1H, *t, J =* 7.69, 14.17)	133.09	—	150.74
5′	—		7.42(1H, *d*, *J* = 8.6)	129.30	7.45 (2H, *t*, *J* = 1.56,5.50,7.38)	128.61	8.19(2H, *d*, *J* = 8.9)	122.98
6′	—		7.95(1H, *d, J =* 8.5)	131.36	8.02 (1H, *d, J =* 7.2)	129.67	8.31(2H,*d, J =* 8.8)	130.80

### 3.4 General Procedure for the Synthesis of Compounds **6–9**

For each reaction, compound **5** (30 mg, 0.086 mmol) and DMAP (31 mg, 0.258 mmol) were dissolved in 2 mL of anhydrous pyridine in a sealed round‐bottom flask under a nitrogen atmosphere at 0°C on a stirrer. Then, benzoyl chloride (24 mg, 0.172 mmol), or paramethylbenzoyl chloride (27 mg, 0.172 mmol), or para‐nitrobenzoyl chloride (32 mg, 0.172 mmol) were added slowly to each solution, separately. After 12 h, each reaction was quenched with 4 mL of 0.2 N hydrochloric acid. The workup was performed in the same manner as previously described, yielding benzoate (compound **6**), paramethylbenzoate (compound **7**), or paranitrobenzoate (compound **8**) ester derivatives of 5 in a pure state. Compound **9**, as an isomer of compound **5** was produced by rearrangement during synthesis as a byproduct (Table 3).

**7α‐O‐para‐chlorobenzoyl‐8β‐methoxy‐1(15),3,11‐cembratriene‐16,2β‐olide (Chlorobenzocembrin, 6)**: colorless oil; 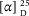
+8.23 (c 0.34, EtOAc); IR (CHCl_3_) ν_max 3425, 2935, 1722, 1595, 1379, 1273, 1093, 1014, 850, 760 cm^−^^1^; UV (CHCl_3_) λ_max (log ε) 278(2.36), 253(3.10); for ^1^H and ^13^C NMR data, see Table 3; HR‐ESI‐MS m/z: 487.226, calcd for C_28_H_38_ClO_5_ + H^+^,487.2245 (Figures S32–S39).

**7α‐O‐benzoyl‐8β‐methoxy‐1(15),3,11‐cembratriene‐16,2β‐olide (Benzocembrin, 7)**: colorless oil; 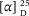
+1637 (c 0.58, EtOAc); IR (CHCl_3_) ν_max 2937, 1732, 1452, 1379, 1275, 1115,1026,758, 714, 667 cm^−^^1^; UV (CHCl_3_) λ_max (log ε) 274.0 (2.19), 258(2.37); for ^1^H and ^13^C NMR data, see Table 3; HR‐ESI‐MS m/z: 453.263, calcd for C_24_H_36_O_5_ + H^+^,453.2635 (Figures S40–S43).

**7α‐O‐paranitrobenzoyl‐8β‐methoxy‐1(15),3,11‐cembratriene‐16,2β‐olide (Nitrobenzocembrin, 8)**: colorless oil; 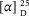
‐44.11 (c 0.17, EtOAc); IR (CHCl_3_) ν_max 2927, 2856, 2355, 1755, 1531, 1454, 1275, 1117, 760, 700 cm^−^^1^; UV (CHCl_3_) λ_max (log ε) 279(1.99), 258.9 (2.78); for ^1^H and ^13^C NMR data, see Table 3; HR‐ESI‐MS m/z: 498.247, calcd for C_2_8H_35_NO7 + H^+^,498.2486 (Figures S44–S47).

**7α‐hydroxy‐8β‐methoxy‐1(15),3,12‐cembratriene‐16,2β‐olide (Trochelioid C, 9)**: colorless oil; 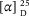
‐14.21 (c 0.57, CHCl_3_); IR (CHCl_3_) ν_max 2962, 2933, 2858, 1755, 1691, 1547, 1452, 1381, 1275, 1171, 1095, 762 cm^−^^1^; UV (CHCl_3_) λ_max (log ε) 256(0.74); ^1^H NMR: δ_H_ 5.56(*bd*, *J* = 10.1, H‐2), 4.93(*d*, *J* = 9.93, H‐3), 2.40(*m*, H‐5), 2.11(*d*, *J* = 7.63, H‐5), 1.45(*m*, H‐6), 1.85(*d*, *J* = 20.54, H‐6), 3.43(*d*, *J* = 11.38, H‐7), 1.83(*d*, *J* = 20.54, H‐9), 1.63(*s*, H‐9), 2.12(*m*, H‐10), 2.12(*m*, H‐11), 2.01(*d*, *J* = 11.13, H‐11), 5.07(*t*, *J* = 7.23, H‐13), 2.76(*m*, H‐14), 2.12(*m*, H‐14), 1.81(*d*, *J* = 1.63, H‐17), 1.84(*d*, *J* = 1.63, H‐18), 1.09(*s*, H‐19), 1.63(*s*, H‐20), 3.17(*s*, 8‐OMe); ^13^C NMR: δ**_C_** 163.00(C‐1), 79.49(C‐2), 120.96(C‐3), 144.22(C‐4), 35.58(C‐5), 26.81(C‐6), 72.80(C‐7), 79.16(C‐8), 30.96(C‐9), 23.37(C‐10), 36.48(C‐11), 134.43(C‐12), 125.46(C‐13), 26.60(C‐14), 122.82(C‐15), 175.03(C‐16), 9.05(C‐17), 16.31(C‐18), 18.56(C‐19), 15.73(C‐20), 49.27(C‐8‐OMe); HR‐ESI‐MS *m/z*: 349.237, calcd. for C_21_H_32_O_4_ + H^+^,349.2373 (Figures S48–S54).

### 3.5 Cell Culture

The MCF‐7 cell line, which is estrogen‐positive for breast cancer (Homo sapiens, female, breast/pleural effusion; ATCCR HTB‐22; RRID: CVCL_0031), along with the MDA‐MB‐231 cell line, which is estrogen‐negative (Homo sapiens, female, breast/pleural effusion; ATCCR HTB‐26; RRID: CVCL_0062), and HUVEC (human umbilical vein endothelial cells; Homo sapiens; umbilical vein endothelium) as non‐malignant normal cells were acquired from the Pasteur Institute located in Tehran, I.R. Iran. Cell lines were maintained under standard conditions (cultured at 37°C in humidified incubators with 5% CO2 and 95% air atmosphere in RPMI‐1640 enriched with 10% heat‐inactivated FBS and 1% penicillin/streptomycin). Cell cultures were checked microscopically to ensure no contamination during the study. Those exhibiting 70%‐80% confluence were passaged using a trypsin/EDTA solution. All treatments were conducted following a 24 h adaptation period for the cells in RPMI‐1640 supplemented with 1% heat‐inactivated FBS and 1% penicillin/streptomycin [15].

### 3.6 Cell Viability by MTT Colorimetric Assay

Cell viability was determined using the MTT method. Cells (7 × 10^3^/well) were seeded in 96‑well plates, incubated overnight, and then treated with test compounds (1‐200 µg/mL) for 48 h. Taxol served as the positive control, and vehicle controls for each dose were included. After treatment, 20 µL of MTT solution (5 mg/mL) was added, followed by a 4 h incubation at 37°C. Formazan crystals were dissolved in 100 µL of DMSO, and absorbance was measured at 570 nm using a BioTek microplate reader. IC_50_ values were calculated from dose–response curves with GraphPad Prism [15].

### 3.7 Flow Cytometry by Annexin V‐FITC/Propidium Iodide Staining

Apoptosis was evaluated using annexin V‑FITC/PI staining according to the manufacturer's protocol. Briefly, 5 × 10^5^ cells per well were seeded in 6‑well plates and allowed to adhere for 24 h, then treated with different concentrations of the tested compound for 48 h. After treatment, cells were washed with cold PBS, centrifuged (1000 g, 5 min), and resuspended in 100 µL of annexin V binding buffer. Following incubation with annexin V (15 min, room temperature, dark) and subsequent PI staining (10 min), samples were analyzed by flow cytometry (FACS Calibur) to determine apoptosis rates [16].

### 3.8 Caspase Activity Assay

Because MCF‑7 cells lack functional caspase‑3 due to a genetic deletion, caspase‑6 was measured as a specific apoptotic marker in this line [17]. Caspase‑6 activity was assessed using a colorimetric kit (R&D Systems, Minneapolis, MN, USA) following the manufacturer's instructions. MCF‑7 and MDA‑MB‑231 cells were seeded overnight and treated with the tested compound at concentrations of 1, 10, 20, 100, and 200 µM for 48 h. After treatment, cells were harvested, lysed in the supplied buffer, and protein extracts were prepared. Enzymatic activity was quantified using a caspase‑6‑specific substrate conjugated to p‑nitroaniline (pNA), and absorbance was measured at 405 nm with a microplate reader [18].

### 3.9 Intracellular ROS Production Assay

Reactive oxygen species (ROS) generation was measured using the fluorogenic probe 2′,7′‑dichlorofluorescein diacetate (DCFDA) following the manufacturer's protocol (Marker Gene Live Cell Co). Cells (4 × 10^3^/well) were seeded in optically clear 384‑well plates and allowed to adhere for 24 h. They were then treated with serial dilutions of the tested compound (1–100 µM) for 48 h. After treatment, the buffer containing DCFDA (20 µM, pH 7.4) was replaced with fresh culture medium, and cells were incubated at 37°C in the dark for 30 min. Fluorescence intensity was recorded at 485/528 nm (excitation/emission) using a Synergy HT Multi‑Mode Microplate Reader [18, 19].

### 3.10 Acute Toxicity Study

Male mice were obtained from the Experimental Animal Center of Isfahan University of Medical Sciences (Ethical approval: IR.MUI.RESEARCH.REC140.148). Animals were maintained under standard laboratory conditions (12 h light/dark cycle, 24°C  ±  3 °C) with free access to sterilized food and water. Before dosing, all mice were fasted for 16 h. The tested compound was prepared in polyethylene glycol containing Pharmasolve 5% as a vehicle. Six treatment groups were established: control (vehicle only) and test groups receiving a single dose of 125, 250, and 500 mg/kg intraperitoneally. Following administration, animals were observed continuously for 48 h to monitor behavioral changes, clinical signs, and mortality. Body weight and general health were recorded throughout 14 days. On day 15, all mice were euthanized with an overdose of ketamine/xylazine. Following the autopsy, liver and kidney tissues were collected for microscopic evaluation. They were fixed in 10% formalin, dehydrated, embedded in paraffin, and sectioned at 5 µm thickness. The prepared slides were stained with hematoxylin and eosin (H&E) and examined under a light microscope to assess structural alterations [15].

### 3.11 Statistical Analyses

The results are expressed as the mean ± standard deviation (SD) derived from three independent experiments. Statistical comparisons between groups were conducted using one‐way analysis of variance and Dunnett's test, as implemented in GraphPad Prism 8.0.1 software (GraphPad Software, La Jolla, CA, USA). A P‐value of less than 0.05 was deemed statistically significant.

## 4 Conclusion

The present investigation demonstrated that *Sarcophyton tenuispiculatum* is a rich source of cembranoid diterpenes. Semi‐synthetic modification of 7α,8α‐epoxy‐1(15),3,11‐cembratriene‐16,2β‐olide (**1**) yielded novel arylated derivatives, among which chlorobenzocembrin (**6**) exhibited more cytotoxicity against breast cancer cell lines. Mechanistic studies revealed that its pro‐apoptotic activity is mediated through elevated intracellular ROS and caspase‐6 activation. These findings are consistent with the established role of structural features in driving apoptogenic activity among natural products. MTT results highlighted that epoxide ring opening and aryl substitution at C7 significantly enhanced cytotoxic activity potential compared to parent compounds. In vivo acute toxicity studies further indicated hepatic and renal damage at doses more than 250 mg/kg, underscoring the need for cautious dose optimization. Altogether, this study identifies cembrane lactones as promising leads for anticancer drug discovery.

## Author Contributions

**Samin Mousavi**: writing – original draft, investigation. **Afsaneh Yegdaneh**: resources, supervision. **Mahmoud Aghaei**: supervision, validation. **Mustafa Ghanadian**: supervision, resources, writing, review and editing.

## Declaration of using AI‐assisted technology in the writing process

During the writing of the text, one of the authors, “MG,” used Grammarly AI only to refine the English language. After using this tool/service, the authors reviewed and edited the text again and accepted full responsibility for the content.

## Conflicts of Interest

The authors declare no conflicts of interest.

## Supporting information




**Supporting File**: cbdv71842‐sup‐0001‐SuppMat.docx.

## Data Availability

The data that support the findings of this study are available on request from the corresponding author. The data are not publicly available due to privacy or ethical restrictions.
