# Supplementary material for: Isolated Cembranoid Diterpenes From Sarcophyton tenuispiculatum and Semi‑Synthetic Derivatives with Pro‑Apoptotic Activity in Breast Cancer Cells
Source: Chem Biodivers. 2026 Oct 15;23(10):e71842. doi: 10.1002/cbdv.71842 (PMC13670404; doi:10.1002/cbdv.71842)
Supplement: Supplementary file 1 [file CBDV-23-e71842-s001.docx]

**Supplementary Information**

*Supplementary materials of the article:*

**Isolated cembranoid diterpenes from *Sarcophyton tenuispiculatum* and semi‑synthetic derivatives with pro‑apoptotic activity in breast cancer cells**

Samin Mousavi^a^, Afsaneh Yegdaneh^a^, Mahmoud Aghaei^b^, Mustafa Ghanadian^c, *^

*^a^Department of Pharmacognosy, School of Pharmacy and Pharmaceutical Sciences, Isfahan University of Medical Sciences, Isfahan, I.R., Iran.*

*^b^Department of Clinical Biochemistry, School of Pharmacy and Pharmaceutical Sciences, Isfahan University of Medical Sciences, Isfahan, I.R., Iran.*

*^c^Isfahan Pharmaceutical Sciences Research Center, Isfahan University of Medical Sciences, Isfahan, I.R., Iran*

*Authors for correspondence: M. Ghanadian (Tel: +98-913316-7326; E-mail: ghannadian@gmail.com)

[Title: Spectroscopic Data for Compounds 1–9 Isolated from](#_Toc210316140) *Sarcophyton tenuispiculatum*

[Figure S1. ^1^H NMR (400 MHz, CDCl3) spectrum of compound 1.	4](#_Toc210316141)

[Figure S2. ^13^C NMR (100 MHz, CDCl3) spectrum of compound 1.	5](#_Toc210316142)

[Figure S3. DEPT-90 spectrum of compound 1.	6](#_Toc210316143)

[Figure S4. DEPT-135 spectrum of compound 1.	7](#_Toc210316144)

[Figure S5. HSQC spectrum of compound 1.	8](#_Toc210316145)

[Figure S6. HMBC spectrum of compound 1.	9](#_Toc210316146)

[Figure S7. NOESY spectrum of compound 1.	10](#_Toc210316148)

[Figure S8. ^1^H NMR(400 MHz, CDCl3) spectrum of compound 2.	11](#_Toc210316151)

[Figure S9. ^13^C NMR (100 MHz, CDCl3) spectrum of compound 2.	12](#_Toc210316152)

[Figure S10. DEPT-90 spectrum of compound 2.	13](#_Toc210316153)

[Figure S11. DEPT-135 spectrum of compound 2.	14](#_Toc210316154)

[Figure S12. HSQC spectrum of compound 2.	15](#_Toc210316155)

[Figure S13. HMBC spectrum of compound 2.	16](#_Toc210316156)

[Figure S14. NOESY spectrum of compound 2.	17](#_Toc210316158)

[Figure S15. ^1^H NMR (400 MHz, CDCl3) spectrum of compound 3.	18](#_Toc210316159)

[Figure S16 ^13^C NMR (100 MHz, CDCl3) spectrum of compound 3.	19](#_Toc210316160)

[Figure S17. DEPT-90 spectrum of compound 3.	20](#_Toc210316161)

[Figure S18. DEPT-135 spectrum of compound 3.	21](#_Toc210316162)

[Figure S19. HSQC spectrum of compound 3.	22](#_Toc210316163)

[Figure S20. HMBC spectrum of compound 3.	23](#_Toc210316164)

[Figure S21. ^1^H NMR(400 MHz, CDCl3 spectrum of compound 4.	24](#_Toc210316169)

[Figure S22. ^13^C NMR (100 MHz, CDCl3) spectrum of compound 4.	25](#_Toc210316170)

[Figure S23. DEPT-90 spectrum of compound 4.	26](#_Toc210316171)

[Figure S24. DEPT-135 spectrum of compound 4.	27](#_Toc210316172)

[Figure S25. ^1^H NMR (400 MHz,CDCl3) spectrum of compound 5.	28](#_Toc210316141)

[Figure S26. ^13^C NMR (100 MHz, CDCl3) spectrum of compound 5.	29](#_Toc210316142)

[Figure S28. HSQC spectrum of compound 5.	30](#_Toc210316145)

[Figure S28. HMBC spectrum of compound 5 .	31](#_Toc210316146)

[Figure S29.](#_Toc210316148) [NOESY spectrum of compound 5.	31](#_Toc210316148)

[Figure S30.](#_Toc210316149) [ESI-MS spectrum of compound 5.	32](#_Toc210316149)

Figure S31. IR spectrum of compound5.	33

Figure S32. ^1^H NMR(400 MHz, CDCl3) spectrum of compound 6.	34

Figure S33. ^13^C NMR (100 MHz, CDCl3) spectrum of compound 6.	35

[Figure S34. DEPT-90spectrum of compound 6.	36](#_Toc210316155)

[Figure S35. DEPT-135spectrum of compound 6.	37](#_Toc210316155)

[Figure S36. HSQC spectrum of compound 6.	38](#_Toc210316155)

[Figure S37. HMBC spectrum of compound 6.	39](#_Toc210316156)

Figure S38. ESI-MS spectrum of compound 6.	40

Figure S39. IR spectrum of compound 6	[41](#_Toc210316149)

Figure S40. ^1^H NMR (400 MHz, CDCl3) spectrum of compound 7.	42

Figure S41. ^13^C NMR (100 MHz, CDCl3) spectrum of compound 7.	43

[Figure S42. ESI-MS spectrum of compound 7.	44](#_Toc210316167)

Figure S43. IR spectrum of compound 7	[45](#_Toc210316149)

Figure S44. ^1^H NMR(400 MHz, CDCl3) spectrum of compound 8.	46

Figure S45. ^13^C NMR (100 MHz, CDCl3) spectrum of compound 8.	47

[Figure S46. ESI-MS spectrum of compound 8.	48](#_Toc210316167)

Figure S47. IR spectrum of compound 8	[49](#_Toc210316149)

Figure S48. ^1^H NMR(400 MHz, CDCl3) spectrum of compound 9.	50

Figure S49. ^13^C NMR (100 MHz, CDCl3) spectrum of compound 9.	51

[Figure S50. HSQC spectrum of compound 9.	52](#_Toc210316173)

[Figure S51. HMBC spectrum of compound 9.	53](#_Toc210316174)

[Figure S52. NOESY spectrum of compound 5.	54](#_Toc210316148)

[Figure S53. ESI-MS spectrum of compound 9.	55](#_Toc210316167)

Figure S54. IR spectrum of compound 9	56

## **Fig.S1.** ^1^H NMR (400 MHz, CDCl3) spectrum of compound 1.

## **Fig.S2.** ^13^C NMR (100 MHz, CDCl3) spectrum of compound 1

## **Fig.S3.** DEPT-90 spectrum of compound 1

## **Fig.S4.** DEPT-135 spectrum of compound 1

## **Fig.S5.** HSQC spectrum of compound 1

## **Fig.S6.** HMBC spectrum of compound 1

## **Fig.S7.** NOESY spectrum of compound 1

## **Fig.S8.** ^1^H-NMR (400 MHz,CDCl3) spectrum of compound 2

## **Fig. S9.** ^13^C-NMR (100 MHz, CDCl3) spectrum of compound 2

## **Fig.S10.** DEPT-90 spectrum of compound 2

## **Fig.S11.** DEPT-135 spectrum of compound 2

## **Figure S12.** HSQC spectrum of compound 2

## **Fig.S13.** HMBC spectrum of compound 2

## **Fig.S14.** NOESY spectrum of compound 2

## **Fig.S15.** ^1^H NMR (400 MHz,CDCl3) spectrum of compound 3

## **Fig.S16.** ^13^C NMR (100 MHz, CDCl3) spectrum of compound 3

## **Fig.S17.** DEPT-90 spectrum of compound 3

## **Fig.S18.** DEPT-135 spectrum of compound 3

## **Fig. S19.** HSQC spectrum of compound 3

## **Fig.S20.** HMBC spectrum of compound 3

## **Fig.S21.** ^1^H NMR(400 MHz, CDCl3) spectrum of compound 4

## **Fig.S22.** ^13^C NMR (100 MHz, CDCl3) spectrum of compound 4

## **Fig.S23.** DEPT-90 spectrum of compound 4

## **Fig.S24.** DEPT-135 spectrum of compound 4.

**Fig.S25**. ^1^H NMR (400 MHz,CDCl3) spectrum of compound 5

**Fig.S26.** 13C NMR (100 MHz, CDCl3) spectrum of compound 5

**Fig.S27.** HSQC spectrum of compound 5

**Fig.S28.** HMBC spectrum of compound 5

**Fig.S29.** NOESY spectrum of compound 5

**Fig.S30.** ESI-MS spectrum of compound 5

**Fig.S31.** IR spectrum of compound 5

**Fig.S32***.* 1H NMR (400 MHz,CDCl3) spectrum of compound 6

**Fig.S33.** ^13^C NMR (100 MHz, CDCl3) spectrum of compound 6

**Fig,S34.** DEPT-90 spectrum of compound 6

**Fig,S35.** DEPT-135 spectrum of compound 6

**Fig.S36**. HSQC spectrum of compound 6

**Fig.S37.** HMBC spectrum of compound **6**

**Fig.S38.** ESI-MS spectrum of compound 6

**Fig.S39.** IR spectrum of compound 6

**Fig.S40***.* ^1^H NMR (400 MHz,CDCl3) spectrum of compound 7

**Fig.S41.** ^13^C NMR (100 MHz, CDCl3) spectrum of compound 7

**Fig.S42.** ESI-MS spectrum of compound 7

**Fig.S43.** IR spectrum of compound 7

**Fig.S44**.^1^H NMR (400.15 MHz) spectrum of compound 8

**Fig.45.**^13^C NMR (100.6 MHz) spectrum of compound 8

**Fig.S46.** ESI-MS spectrum of compound8.

**Fig.S47.** IR spectrum of compound 8

**Fig.S48**.^1^H NMR (400.15 MHz) spectrum of compound 9

**Fig.S49**.^13^C NMR (100.6 MHz) spectrum of compound **9**

**Fig.S50.** HSQC spectrum of compound 9

**Fig.S51.** HMBC spectrum of compound **9**

**Fig.S52.** NOESY spectrum of compound **9**

**Fig.S53.** ESI-MS spectrum of compound 9.

**Fig.S54.** IR spectrum of compound 9
